# Staphylococcal Cassette Chromosome *mec* (SCC*mec*) Natural Excision Frequencies and Its Contributing Factors in Variant SCC*mec* Type Prototypic Strains

**DOI:** 10.3390/antibiotics15060555

**Published:** 2026-05-30

**Authors:** Salman Mirza, Laura Fine, Jo-Ann McClure, Joseph Kim, John M. Conly, Kunyan Zhang

**Affiliations:** 1 Department of Pathology & Laboratory Medicine, University of Calgary and Alberta Health Services, Calgary, AB T2N 4N1, Canada; 2 Department of Microbiology, Immunology and Infectious Diseases, University of Calgary, Calgary, AB T2N 4N1, Canada; 3 Centre for Antimicrobial Resistance, Alberta Health Services/Alberta Precision Laboratories/University of Calgary, Calgary, AB T2N 4N1, Canada; 4 Department of Medicine, University of Calgary and Alberta Health Services, Calgary, AB T2N 4N1, Canada; 5 The Calvin, Phoebe and Joan Snyder Institute for Chronic Diseases, University of Calgary and Alberta Health Services, Calgary, AB T2N 4N1, Canada

**Keywords:** Methicillin-Resistant *Staphylococcus aureus* (MRSA), SCC*mec* excision, staphylococcal cassette chromosome *mec*, mobile genetic elements, antimicrobial resistance, quantitative PCR, environmental stress, β-lactam antibiotics, genomic plasticity, bacterial evolution

## Abstract

Background: *Staphylococcus aureus* acquires methicillin resistance genes through the SCC*mec* element. Although spontaneous SCC*mec* excision has been observed, its frequency, type-specific variation, and responsiveness to environmental conditions remain undefined. Here, we systematically quantified SCC*mec* excision across diverse prototypic types/subtypes and evaluated the factors that contribute to excision variability. Methods: Twenty five prototypic MRSA strains (SCC*mec* types I–VIII, XI–XIII and defined subtypes) were examined under standard growth temperature (37 °C), elevated temperature (42 °C), desiccation, prolonged continuous culture (30 days), and sub-lethal oxacillin pressure. Excision frequencies were quantified using qPCR, normalized to the *gyrB* housekeeping gene using the formula: 10^−((*Ct,orfX*−*Ct,gyrB*)/3.32)^. Statistical analyses included one-way ANOVA, *t*-tests, and OLS regression for time-dependent trends. Results: At 37 °C, excision frequencies ranged from 2.40 × 10^−6^ to 1.32 × 10^−3^ and varied among representative SCC*mec* types/subtypes but were unrelated to SCC*mec* size (*R*^2^ = 0.027, *p* = 0.44). Type I showed no detectable excision due to a truncated *ccrB* gene. At 42 °C, excision increased in 14 of 24 types (median +11.2%; eight significant) and decreased in 10 (median −7.4%; four significant). Desiccation produced similar effects, with nine types increasing (median +7.1%; four significant), 14 decreasing (median −8.2%; five significant), and one unchanged. Continuous culture exhibited progressive increases in excision across multiple types (*R*^2^ = 0.3–0.94), whereas sub-lethal oxacillin uniformly maintained low detectable excision frequencies across all SCC*mec* types. Conclusions: Excision varied among representative SCC*mec* types and was influenced heterogeneously by distinct stress conditions. Continuous culture promoted excision, whereas oxacillin exposure maintained low detectable excision. This work quantitatively confirms spontaneous SCC*mec* excision and provides new insights into MRSA genome plasticity.

## 1. Introduction

*Staphylococcus aureus* is a versatile opportunistic pathogen capable of causing illnesses ranging from minor skin and soft-tissue infections to severe invasive diseases such as bacteremia, pneumonia, and endocarditis [1,2,3]. The emergence and global spread of methicillin-resistant *S. aureus* (MRSA) has greatly complicated clinical care, as MRSA strains exhibit resistance to nearly all β-lactam antibiotics [4]. This resistance contributes to increased morbidity, mortality, and healthcare burden in both community and hospital settings [5].

Methicillin resistance in *S. aureus* is carried on the staphylococcal cassette chromosome *mec* (SCC*mec*), a mobile genetic element that integrates site-specifically into the chromosomal *orfX* locus [6,7,8,9]. Central to this element is the *mec* gene complex, most commonly *mecA*, which encodes the low-affinity penicillin-binding protein PBP2a that preserves cell-wall synthesis under β-lactam exposure [4,10,11,12]. A less common homolog, *mecC*, shares ~70% nucleotide identity with *mecA* but produces a biochemically distinct yet functionally similar PBP2a variant [6,13,14,15]. Surrounding the *mec* complex is a modular genomic architecture defined primarily by the cassette chromosome recombinase (*ccr*) gene complex and the joining (J) regions. The *ccr* genes (*ccrA*/*ccrB*, or *ccrC*) encode the recombinases that mediate precise excision and integration at the chromosomal *orfX* locus [6,16,17]. This recombination reaction occurs between the chromosomal *attB* site at *orfX* and the cassette-borne *attSCC* site, generating the direct repeat junctions (DR-L and DR-R) that characterize SCC*mec* insertion and excision [7,18,19]. The flanking J-regions, by contrast, are highly variable and encode accessory genes that contribute to cassette heterogeneity [6]. Variation in the architecture of the *ccr* complex, together with the organization of the *mec* gene complex and the highly variable J regions, underlies the classification of SCC*mec* elements into distinct types and subtypes [6,20]. To date, fifteen SCC*mec* types (I–XV) and numerous subtypes have been described, each defined by characteristic combinations of these structural features [6,21,22]. This structural diversity reflects the evolutionary plasticity of SCC*mec* and highlights its central role in shaping the resistance and adaptability of MRSA lineages.

While this structural framework provides a thorough basis for characterizing SCC*mec* diversity, it also raises key questions about how these elements function within the chromosome, especially regarding their capacity to excise.

Early work by Ito et al. [23] first demonstrated that SCC*mec* can excise from the chromosome in strain N315 (SCC*mec* type II). Using a highly sensitive nested-PCR assay targeting the reconstituted chromosomal *attB* junction, they detected precise loss of *mec* DNA at a low frequency (<10^−4^), providing the earliest molecular evidence that SCC*mec* excision is a phenomenon that can occur. Subsequent studies further confirmed the mechanistic basis of this process, demonstrating that SCC*mec* and related mobile genetic elements, such as ACME, can be mobilized through CcrAB-mediated site-specific recombination, resulting in precise chromosomal excision and formation of circular intermediates [24]. Clinical evidence of in vivo SCC*mec* loss was reported by Boundy et al. [25], who identified isogenic MRSA–MSSA nasal-carrier pairs from the same individuals. By comparing PFGE patterns, *spa* types, and PCR amplification of the *attB* junction, they demonstrated that MSSA isolates had lost SCC*mec* while retaining an identical chromosomal background, consistent with naturally occurring excision events. Building on these observations, Stojanov et al. quantified SCC*mec* excision directly in N315 using a sensitive qPCR assay targeting the reconstituted *attB* site, estimating an excision frequency of ~2 × 10^−6^ [26]. Together, these studies confirm that spontaneous SCC*mec* excision is indeed a natural and measurable phenomenon in MRSA.

Here, we extend a widely used quantitative PCR–based framework for detecting recombined chromosomal junctions formed after SCC*mec* excision, normalized to a chromosomal reference gene [27,28,29,30,31]. Notably, Wu et al. from our laboratory previously applied and experimentally validated this framework in the USA300 lineage to quantify excision of both SCC*mec* and the arginine catabolic mobile element (ACME) [32].

Despite these foundational advances, previous work has largely focused on a small number of strains and experimental conditions. Therefore, it remains unclear whether SCC*mec* excision occurs broadly across diverse SCC*mec* types, how diverse environmental stressors affect this process, and whether different SCC*mec* backgrounds exhibit conserved or divergent excision responses. The ecological, environmental and strain-specific factors contributing to SCC*mec* excision variability therefore remain undefined.

In this study, we apply this established quantitative framework across a broader panel of 25 prototypic MRSA strains representing SCC*mec* types I–VIII and XI–XIII, including major subtypes. We quantified baseline excision frequencies under standard growth conditions and assessed how heat stress, desiccation, prolonged continuous culture, and sub-lethal β-lactam exposure shape excision dynamics across this diverse panel. These conditions were selected to model physiologically relevant stressors associated with bacterial growth, environmental persistence, and β-lactam selective pressure. Our findings show that SCC*mec* excision is a widespread phenomenon, influenced distinctly by environmental conditions, and likely additional strain-specific determinants, revealing new insights into the acquisition and dissemination of methicillin resistance in *S. aureus*.

## 2. Results

### 2.1. SCCmec Excision Exhibits Type-Specific Variation but Not Associated with SCCmec Size, Under Optimal Growth Condition (37 °C)

**Figure 1:**
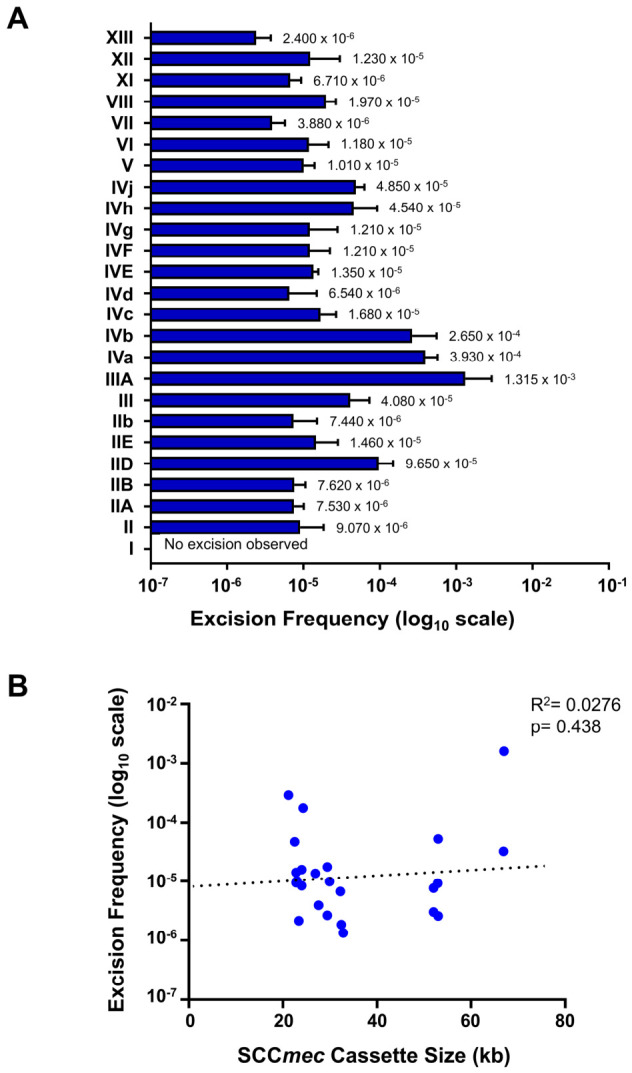
Baseline SCC*mec* excision frequencies and size–excision relationship under standard growth conditions (37 °C). (**A**) Excision was quantified by real-time qPCR using primers specific to the recombined SCC*mec*-*orfX* junction and normalized to the housekeeping gene *gyrB*. Bars show mean log_10_ excision frequency ± SD for each SCC*mec* type/subtype (*n* = 3 biological replicates). SCC*mec* type I (NCTC10442) showed no detectable excision and was excluded from statistical analyses. (**B**) Linear regression analysis assessing the relationship between SCC*mec* cassette size (kb) and corresponding excision frequency across the MRSA strains tested. Blue dots represent individual *SCCmec* types/subtypes, and the dotted line indicates the linear regression fit.

Under standard growth conditions (37 °C), SCC*mec* excision was detected in 24 of 25 MRSA prototypic strains representing (SCC*mec* types I–VIII and XI–XIII, including defined subtypes) (Figure 1A). Type I showed no detectable excision. Among excision-competent strains, excision frequencies spanned nearly three orders of magnitude, ranging from ~10^−3^ to ~10^−6^ across the panel. Type II displayed a lower baseline value (9.07 × 10^−6^), similar to the closely related variants IIA, IIB, and IID. Type IIE excised at 1.46 × 10^−5^, while types III and IIIA showed higher baseline values, including 1.32 × 10^−3^ for IIIA. Excision frequencies for SCC*mec* IV subtypes (IVa, IVb, IVc, IVd, IVe, IVF, IVg, IVh, IVj) ranged from 1.21 × 10^−5^ to 3.93 × 10^−4^. Types V, VI, VII, and VIII showed frequencies ranging between 3.88 × 10^−6^ and 1.97 × 10^−5^. Finally, types XI, XII, and XIII exhibited low but measurable excision, ranging from 2.40 × 10^−6^ to 1.23 × 10^−5^.

SCC*mec* type accounted for a substantial proportion of the variation in excision frequency, with one-way ANOVA showing a highly significant effect (*F*(23,48) = 8.92, *p* = 1.3 × 10^−10^; *R*^2^ = 0.8104). By comparison, cassette size showed no relationship with excision frequency, as indicated by linear regression (*R*^2^ = 0.0276, *p* = 0.438) (Figure 1B).

### 2.2. SCCmec Excision Shows Heterogeneity Under Heat Stress (42 °C) and Desiccation with Type-Specific Variation

Exposure to elevated temperature (42 °C) produced heterogeneous and type-specific changes in excision, with variation in both magnitude and direction. Relative to 37 °C, excision frequency increased in 14 SCC*mec* types (range: +0.8% to +28.0%; median increase: 11.2%), eight of which showed significant within-type induction (*p* ≤ 0.05). Ten types instead showed reduced excision at 42 °C (range: −0.2% to −33.4%; median decrease: 7.4%), including four with significant decreases (*p* ≤ 0.05).

**Figure 2:**
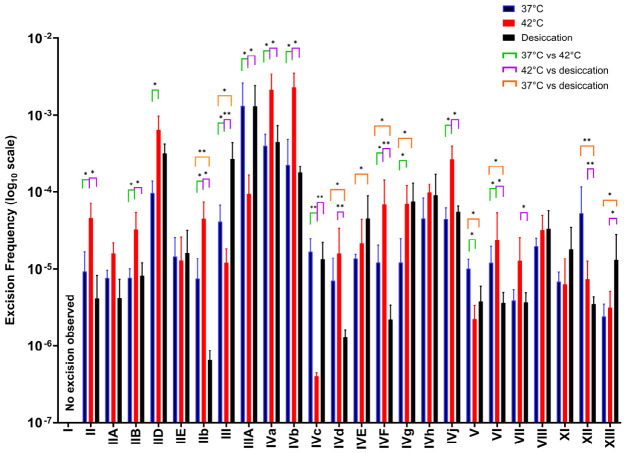
SCC*mec* excision responses to heat stress and desiccation across diverse SCC*mec* types I–VIII and XI–XIII (including subtypes). MRSA strains were incubated under standard conditions (37 °C; blue), heat stress (42 °C; red), or desiccation (25 °C, 6 days; black). SCC*mec* excision frequency was quantified by real-time qPCR targeting the SCC*mec*-*orfX* junction and normalized to *gyrB*. Bars indicate log_10_ mean excision frequency ± SD (*n* = 3 independent biological replicates). Brackets denote paired within-strain comparisons: green, 37 °C vs. 42 °C; purple, 42 °C vs. desiccation; orange, 37 °C vs. desiccation. Asterisks indicate statistical significance from paired two-tailed *t*-tests (* *p* ≤ 0.05, ** *p* ≤ 0.01). SCC*mec* type I showed no excision above the assay limit of detection under any condition.

Likewise, desiccation stress (25 °C, 6 days) showed no consistent overall trend in excision, with responses varying by SCC*mec* type. Excision frequency increased in nine SCC*mec* types (range: +1.0% to +18.0%; median increase: 7.1%), with four reaching statistical significance (*p* ≤ 0.05). Fourteen types showed decreased excision (range: −0.2% to −17.7%; median decrease: 8.2%), five of which were significant (*p* ≤ 0.05), and one with no detectable change (Figure 2).

Across the entire strain panel, mean log_10_ excision frequencies (± SD) were 4.79 ± 0.64 at 37 °C, 4.67 ± 0.91 at 42 °C, and 4.88 ± 0.88 under desiccation. Pairwise comparisons revealed no overall significant difference between 37 °C and 42 °C (*p* = 0.47) or between 37 °C and desiccation (*p* = 0.72).

Linear regression revealed no association between SCC*mec* size and excision under either stress conditions. Under heat stress, size explained 0.24% of the variance (*R*^2^ = 0.0024; *slope* = −0.00299; *p* = 0.8208). Under desiccation, size explained 4.62% of the variance (*R*^2^ = 0.046; *slope* = −0.01266; *p* = 0.3129) (see Figure S1).

### 2.3. Descriptive Percentile-Based Grouping of SCCmec Excision Frequencies to Guide Representative Strain Selection for Continuous Culture and Antibiotic Pressure Studies

**Figure 3:**
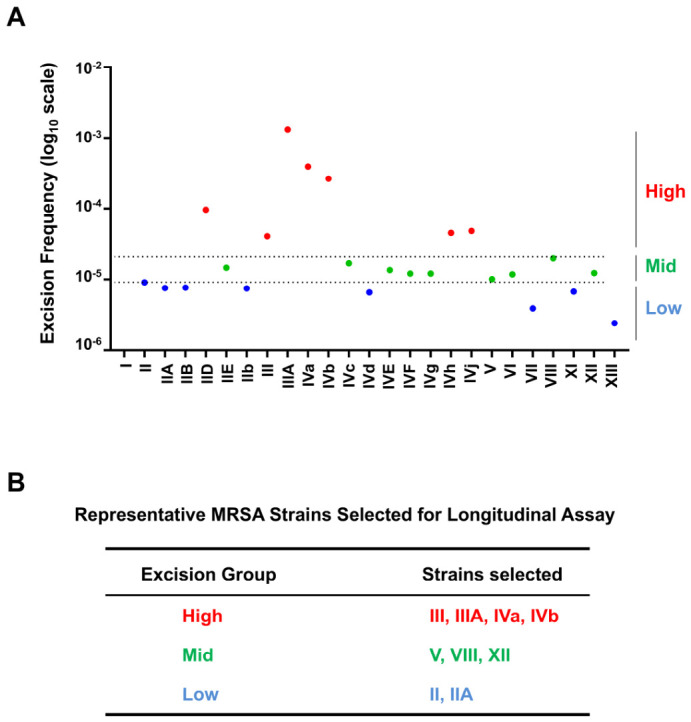
Percentile-based stratification of SCC*mec* types and representative strain selection based on excision frequencies under optimal growth conditions (37 °C). (**A**) Baseline SCC*mec* excision frequencies were quantified by real-time qPCR targeting the *orfX* junction and normalized to *gyrB.* Values are plotted on a log_10_ scale and grouped into three descriptive excision categories based on percentile rank: high (top third; red), mid (middle third; green), and low (bottom third; blue). Dotted horizontal lines indicate the 33rd and 67th percentile cut-offs. (**B**) Representative MRSA strains selected from each excision group for 30-day continuous culture and sub-lethal oxacillin pressure assays.

To capture the range of baseline SCC*mec* excision for representative strain selection, all 24 excision-competent SCC*mec* types were ranked by their mean excision at 37 °C and stratified into descriptive percentile-based categories (Figure 3A). Types with mean excision frequencies above ~10^−4.7^ were classified as high-excision (*n* = 7), corresponding to the upper threshold demarcation in Figure 3A. Types with values between ~10^−5.1^ and ~10^−4.7^ were assigned to the mid-excision group (*n* = 9). Types below ~10^−5.1^ were categorized as low excision (*n* = 8), occupying the region below the lower dashed threshold. These categories were used solely to guide representative strain selection for longitudinal experiments.

Phenotypic oxacillin resistance was confirmed for all isolates using a Kirby–Bauer disk diffusion assay (see Table S1), verifying that each strain maintained the expected MRSA resistance profile under the subsequent long-term assay conditions. For oxacillin stress condition, cultures were exposed to sub-lethal oxacillin (2 μg/mL), selected based on CLSI susceptibility breakpoints (≥4 μg/mL for MRSA) to impose β-lactam pressure without inhibiting growth [33,34].

For longitudinal analyses, the high-excision group was represented by SCC*mec* types III, IIIA, IVa, and IVb; the mid-excision group by types V, VIII, and XII; and the low-excision group by types II and IIA (Figure 3B). Together, this panel encompassed the full spectrum of baseline excision phenotypes for subsequent continuous culture and β-lactam exposure experiments.

### 2.4. Continuous Culture Promotes Excision with Type-Specific Variation While Antibiotic Pressure Inhibits Excision

#### 2.4.1. High-Excision Group (Types III, IIIA, IVa, IVb)

**Figure 4:**
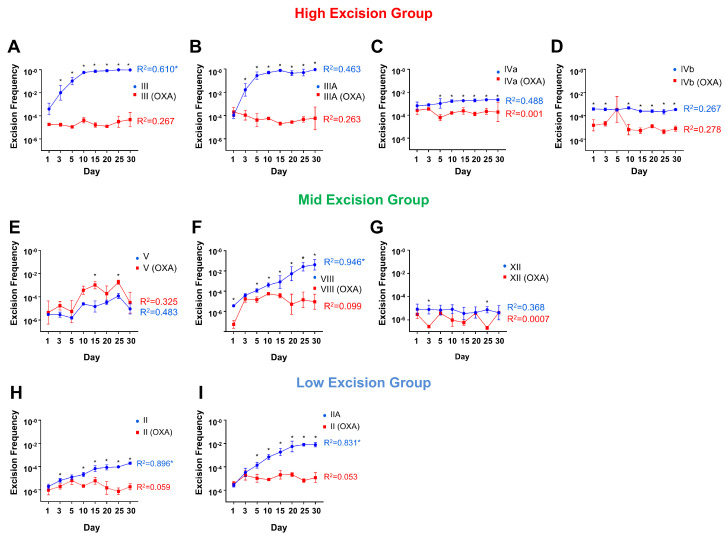
Excision dynamics of SCC*mec*-type MRSA strains during 30-day continuous culture and sub-lethal oxacillin pressure. Representative MRSA strains from the high-excision group included (**A**) SCC*mec* type III, (**B**) type IIIA, (**C**) type IVa, and (**D**) type IVb. Representative strains from the mid-excision group included (**E**) type V, (**F**) type VIII, and (**G**) type XII. Representative strains from the low-excision group included (**H**) type II and (**I**) type IIA. Strains were serially passaged for 30 days either in antibiotic-free BHI medium (continuous culture; blue) or under sub-lethal oxacillin (2 µg/mL) pressure (OXA; red). Excision frequencies were quantified over 30 days by qPCR and plotted on a log_10_ scale as mean ± SEM of three biological replicates. Solid lines show linear regression fits with corresponding *R*^2^ values. Asterisks indicate significant differences between continuous culture and oxacillin-treated conditions at individual time points (* *p* < 0.05, two-tailed unpaired *t*-test).

High-excision SCC*mec* types maintained elevated excision frequencies and showed variable increases during continuous culture. SCC*mec* type III increased rapidly during the first 10 days before plateauing (*R*^2^ = 0.610, *p* = 0.022), whereas oxacillin-treated cultures maintained low excision frequencies with no detectable trend (*R*^2^ = 0.267, *p* = 0.190). Type IIIA showed a similar upward pattern that approached significance (*R*^2^ = 0.463, *p* = 0.063), with no detectable temporal change under oxacillin exposure (*R*^2^ = 0.263, *p* = 0.194). SCC*mec* type IVa showed no significant temporal change during continuous culture (*R*^2^ = 0.488, *p* = 0.054) and maintained low stable excision frequencies under oxacillin exposure (*R*^2^ = 0.001, *p* = 0.938). SCC*mec* type IVb showed stable excision under both conditions (continuous culture: *R*^2^ = 0.267; oxacillin: *R*^2^ = 0.278). Significant differences between conditions emerged between days 3–5 (Figure 4A–D).

#### 2.4.2. Mid-Excision Group (Types V, VIII, XII)

Mid-excision SCC*mec* types showed heterogeneous responses to continuous culture. SCC*mec* type V exhibited modest fluctuations with a non-significant upward trend (*R*^2^ = 0.483, *p* = 0.056), while oxacillin-exposed cultures maintained low stable excision frequencies with no significant temporal change (*R*^2^ = 0.325, *p* = 0.140). SCC*mec* type VIII increased strongly during continuous passage (*R*^2^ = 0.946, *p* = 5.15 × 10^−5^), whereas oxacillin-treated cultures maintained low stable excision frequencies (*R*^2^ = 0.099, *p* = 0.447). SCC*mec* type XII displayed no significant trend under either condition (continuous culture: *R*^2^ = 0.368, *p* = 0.111; oxacillin: *R*^2^ = 0.0007, *p* = 0.949). Significant differences between continuous culture and oxacillin were observed at selected time points depending on the strain (Figure 4E–G).

#### 2.4.3. Low-Excision Group (Types II and IIA)

Low-excision SCC*mec* types showed strong time-dependent increases during continuous culture. For SCC*mec* type II, excision frequency increased steadily across the 30-day period (*R*^2^ = 0.896, *p* = 3.7 × 10^−4^), whereas oxacillin-treated cultures maintained consistently low but detectable excision frequencies with no significant temporal change (*R*^2^ = 0.059, *p* = 0.562). Similar behavior was observed for SCC*mec* type IIA, which showed a significant upward trajectory during continuous passage (*R*^2^ = 0.831, *p* = 0.0016) but remained stable under sub-lethal oxacillin pressure (*R*^2^ = 0.053, *p* = 0.583). For both types, excision frequencies diverged significantly between conditions beginning on day 3–5 and remained distinct for the remainder of the experiment (Figure 4H,I).

#### 2.4.4. *R*^2^ Comparison Between Hospital- and Community-Associated SCC*mec* Types

Across all SCC*mec* types, larger hospital-associated (HA-MRSA) cassettes (II, IIA, III, IIIA) showed higher mean regression (mean *R*^2^ = 0.700 ± 0.200) during continuous culture compared with smaller community-associated (CA-MRSA) elements (IVa, IVb, V; mean *R*^2^ = 0.413 ± 0.126). Corresponding individual regression parameters for each strain are provided in Figure S2.

## 3. Discussion

qPCR-based detection of recombination is a widely adopted and experimentally validated approach for quantifying excision of mobile genetic elements in bacterial populations [26,27,28,29,30,31,32,35]. This approach relies on the amplification of recombined chromosomal junctions generated following excision, such as the reconstituted chromosomal *attB* site in *S. aureus*, and normalization of these signals to a chromosomal reference gene to estimate the relative abundance of excision events. Because real-time PCR measurements are inherently exponential, threshold-cycle (Ct) values are interpreted using ΔCt-based transformations consistent with established real-time PCR quantification [27,28,29,30,31]. Within this context, excision is quantified as a normalized, population-level estimate of recombined chromosomal junctions.

This approach has been experimentally validated and applied in MRSA. Stojanov et al. quantified SCC*mec* excision frequency in strain N315 [26]. Diep et al. demonstrated excision of SCC*mec* and the arginine catabolic mobile element (ACME) in USA300 [24]. Boundy et al. identified in vivo SCC*mec* loss through PCR detection of the reconstituted *attB* junction in isogenic MRSA–MSSA nasal-carrier pairs [25]. Previously, in our laboratory, we experimentally validated and applied the technique in MRSA, with excision frequency of both SCC*mec* and ACME quantified in USA300 using *gyrB*-normalized Ct-based principles in conjunction with bacterial plating and counts [32,35]. Together, these findings validate the use of junction-specific PCR approaches as a reliable strategy for assessing SCC*mec* excision at the population level.

In this context, SCC*mec* excision represents a key process governing β-lactam resistance maintenance and genomic plasticity in MRSA [6,36,37]. Although excision has been characterized in the prototypic strain N315 [23,26] and in the epidemic USA300 lineage [32], its prevalence and variability across diverse SCC*mec* types has remained unknown.

To address this gap, we first quantified excision across twenty five prototypic MRSA strains representing SCC*mec* types I–VIII and XI–XIII (including subtypes) under standard growth conditions (37 °C) in nutrient-rich medium, the optimal environment for *S. aureus* growth, which provided a necessary biological baseline against which to interpret subsequent environmental stress conditions [38,39]. Under the standard condition, excision frequencies varied substantially, nearly three orders of magnitude between SCC*mec* types. Larger elements did not excise less frequently, nor did smaller elements exhibit uniformly higher or lower excision, despite the presumed fitness cost associated with maintaining large, and more complex mobile elements [40,41]. The absence of a size–excision relationship indicates that features beyond simply the total cassette size/length underlie the baseline excision variability. Because some SCC*mec* subtypes cassette sizes were derived from published reference genome annotations rather than experimentally reconfirmed in the present study, minor inaccuracies in estimated cassette lengths may influence regression-based interpretation.

To test whether environmental conditions modulate excision, we evaluated SCC*mec* excision dynamics under elevated temperature and desiccation, both of which induce regulatory and physiological changes in *S. aureus* and other bacterial organisms [42,43,44]. Heat activates classical heat-shock pathways [42], including GroEL/GroES [45], σ^B^-dependent stress regulons [46], and perturbs membrane fluidity [47], while enhancing horizontal gene transfer in related organisms [43]. Likewise, desiccation represents a severe environmental stress for *S. aureus*, requiring general stress-response systems for survival. For example, ClpX/ClpXP, SigB, and YjbH have all been shown to contribute to desiccation tolerance [48]. If excision were broadly stress-responsive, these conditions might induce uniform increases or conserved patterns across SCC*mec* types, which would have implications in clinical settings where MRSA is constantly exposed to various stress conditions. Instead, excision responses to both heat and desiccation were highly heterogeneous, with individual SCC*mec* types showing increased, decreased, or unchanged excision relative to baseline. Cassette size again showed no significant effect under either heat (R^2^ = 0.0024) or desiccation (R^2^ = 0.04), arguing against a generalized stress-induced mechanism.

The fact that heat and desiccation are known to induce physiological changes yet fail to produce generalized excision outcomes suggests that environmental stress does not act through a uniform excision-inducing pathway. Instead, additional cassette-intrinsic and/or strain-specific determinants may govern SCC*mec* behavior under both standard and stressed conditions. Variation in *ccr* allotypes (e.g., *ccrA1/B1* versus *ccrA2/B2*), represents one likely contributor [6]. Large serine recombinases (LSR) such as Ccr usually share a conserved catalytic core but differ in auxiliary domains, oligomerization behavior, and DNA-interaction surfaces [49,50]. Such differences reflective in Ccr may influence direct repeat (DR) recombination site recognition, substrate specificity, or the stability of synaptic recombination complexes [51], resulting in distinct excision frequencies. Similarly, polymorphisms at DR sites [52] may influence sequence-specific recognition motifs or alter local DNA curvature, impacting recombination stability and directionality [53]. Additionally, variation in chromosomal context at *orfX*, including differences in DNA topology, nucleoid-associated protein binding, transcriptional activity, and local chromosomal architecture, may modulate recombinase access to DR sites [54]. Together, these structural and genetic factors, or a combination thereof, provide a potential explanation for the variation in excision behaviors observed across different conditions.

Because SCC*mec* encodes the β-lactam resistance determinants that define MRSA, we also examined how long-term nutrient-rich growth versus β-lactam selection influences excision dynamics. Prolonged growth in antibiotic-free medium revealed progressive increases in excision among strains harboring large hospital-associated SCC*mec* elements [6], including types II, IIA, III, and IIIA, indicating that excision remains an active, cumulative process when nutrients are abundant. In contrast, strains carrying smaller community-associated elements (IVa, IVb, V) exhibited stable, low excision frequencies with minimal change. This pattern is consistent with a higher metabolic burden imposed by larger, more complex SCC*mec* elements under long-term propagation [40,55]. Hospital-associated SCC*mec* elements are substantially larger than their community-associated counterparts because they carry additional resistance determinants, heavy-metal tolerance genes, and accessory regulators beyond the *mec* gene complex [6,56]. For example, SCC*mec* type III (~67 kb) contains the *mecA* locus and additional resistance determinants, including aminoglycoside, macrolide, and heavy-metal resistance islands [57,58]. These expanded regions enable survival under the intense selective pressures seen in hospital environments, such as frequent antibiotic exposure, and substantial competition with multi-drug-resistant strains. In contrast, community-associated SCC*mec* elements (e.g., types IVa, IVb, and V) are streamlined, typically 20–28 kb, and encode primarily the *mec* gene complex with fewer additional resistance determinants [59]. Their smaller size reflects adaptation to community settings, where antibiotic pressure is lower and compact elements impose minimal growth cost [59,60]. This ecological and genomic distinction provides a mechanistic context for our continuous culture findings. Larger HA-MRSA cassettes may impose greater fitness costs during long-term growth in nutrient-rich, antibiotic-free conditions, potentially making their loss through excision more favorable. Under such conditions, the selective disadvantage of carrying a large, multi-component SCC*mec* is favored, allowing excision to accumulate progressively over time. In contrast, smaller CA-MRSA cassettes may impose less metabolic costs, remain stable during prolonged growth, and show minimal change in excision frequency. Since direct growth-rate measurements, competition assays, or fitness analyses were not performed, this interpretation remains inferential. Similar size-dependent fitness costs have been demonstrated for conjugative transposons, large pathogenicity islands (~200 kb), and vancomycin-resistance plasmids (80–200 kb) in *Enterococcus*, where newly acquired MGEs can reduce host fitness by 10–30% depending on size, gene content, and integration site [61].

However, under sub-lethal oxacillin exposure, excision remained uniformly low but detectable temporally across all SCC*mec* types throughout the full 30-day experiment. These persistently low detectable excision frequencies under sub-lethal oxacillin exposure are consistent with the theoretical frameworks that strong selection pressures stabilize otherwise burdensome MGEs, overriding metabolic disadvantages to ensure retention of critical functions such as in the case of antibiotic resistance [62,63]. In this context, the selective requirement to maintain the *mecA* gene for β-lactam survival may favor retention of SCC*mec*-containing cells across different cassette backgrounds. However, the present study cannot distinguish between direct physiological inhibition of excision machinery and selective elimination or underrepresentation of excised susceptible cells under oxacillin exposure. Nonetheless, these findings reveal that SCC*mec* mobility is sensitive to its environment and changes dynamically according to the pressure to which it is exposed.

Some considerations should be noted when interpreting these results. Because a single prototypic strain was used to represent each SCC*mec* type/subtype, the observed differences may reflect both SCC*mec-*associated effects and strain-specific genomic variation. Therefore, additional factors such as chromosomal background, regulatory context, and genetic architecture may influence excision behavior independently of SCC*mec* structure. Accordingly, the present findings should be interpreted as representative comparisons across experimental conditions, rather than as universally conserved SCC*mec* type behaviors. Future studies incorporating multiple strains per SCC*mec* type will be required to determine the extent to which these patterns are broadly generalizable. Overall, this study provides novel insights into SC*Cmec* excision and highlights the complexity of excision dynamics in MRSA, shaped by both cassette features and the potentially broader genomic context of the host strain.

## 4. Materials and Methods

### 4.1. Bacterial Strains

Twenty five methicillin-resistant *Staphylococcus aureus* (MRSA) strains representing SCC*mec* types I–VIII and XI–XIII (including defined subtypes) were used in this study. Strains were selected as prototypic representatives of major epidemiological and structural SCC*mec* lineages. Not all currently described SCC*mec* types were included in this study; for example, SCC*mec* types IX, X, and XIV were not examined because the representative strains were not available for inclusion at the time of the study. Metadata, including isolate ID, SCC*mec* type/subtype, source, and genome accession, are provided in Table S2. All strains were maintained as glycerol stocks at −80 °C and streaked onto Tryptic Soy Agar (TSA) before use.

### 4.2. Standard Bacterial Growth Conditions

Unless otherwise stated, cultures were grown overnight in Brain Heart Infusion (BHI) broth at 37 °C with orbital shaking at 200 rpm in 14 mL glass culture tubes (2 mL working volume). Cells from 1 mL culture were harvested for genomic DNA extraction as described below.

### 4.3. Heat Stress and Desiccation Stress Assays

For heat stress assays, cultures were incubated overnight at 42 °C under identical shaking conditions. For desiccation assays, overnight cultures grown at 37 °C were diluted 1:5 in sterile 0.9% NaCl, and 500 µL aliquots were distributed into outer (peripheral) wells of sterile 16-well polystyrene plates. Plates were placed in a sealed incubator at 25 °C for 6 days before rehydration and DNA extraction.

### 4.4. Oxacillin Susceptibility Testing

Oxacillin susceptibility was confirmed for all 25 MRSA strains using standard Kirby–Bauer disk diffusion on Mueller–Hinton agar. Overnight cultures were adjusted to a 0.5 McFarland standard and lawn-inoculated onto agar plates. Oxacillin (1 µg) disks were applied, and plates were incubated at 35 °C for 24 h. Zone diameters were interpreted according to CLSI criteria, <10 mm, resistant; 11–12 mm, intermediate; ≥13 mm, susceptible (see Table S1). All strains used in this study were prototypic MRSA strains based on documented SCC*mec* carriage and strain metadata. Oxacillin disk diffusion was performed simply to assess phenotypic susceptibility under the experimental conditions tested and to guide interpretation of downstream oxacillin-exposure experiments.

### 4.5. Continuous Culture and Antibiotic Pressure Assays

For continuous culture, nine representative strains (spanning high-, mid-, and low-excision classes defined in Figure 3) were serially passaged for 30 days. Each day, 20 µL of culture was transferred into 2 mL fresh BHI (1:100 dilution) and incubated at 37 °C with shaking. Parallel cultures were maintained under sub-lethal β-lactam exposure using BHI supplemented with oxacillin (2 µg/mL), with identical daily passaging. The oxacillin concentration was selected based on CLSI susceptibility breakpoints for MRSA (MIC ≥ 4 µg/mL) and was chosen to impose β-lactam selective pressure while remaining below inhibitory levels for growth [33,34]. Independent tubes were used for each strain and condition to prevent cross-contamination.

### 4.6. Genomic DNA Extraction

Genomic DNA was isolated using the DNeasy Blood & Tissue Kit (Qiagen, Hilden, Germany) with an added staphylococcal lysis step. One milliliter of culture was pelleted (7500 rpm, 10 min), resuspended in AL buffer containing lysostaphin, and incubated for 30 min at 37 °C before proceeding with the manufacturer’s protocol. DNA was eluted in 50 µL nuclease-free water and stored at −20 °C.

### 4.7. Real-Time Quantitative PCR (qPCR) for Quantifying SCCmec Excision

SCC*mec* excision was quantified using a dual-probe qPCR assay targeting the reconstituted chromosomal *attB* junction at *orfX* (marker of precise SCC*mec* excision) together with the chromosomal housekeeping gene *gyrB* for normalization. Primer and probes sequences, including SCC*mec* type-specific *orfX* variants, are listed in Table S3. The SCC*mec* type-to-primer mapping for each strain is provided in Table S4. According to qPCR quantification principles, differences in Ct values reflect relative differences in template abundance [27,28,29,30,31]. Therefore, excision frequencies were normalized to the housekeeping gene *gyrB* and interpreted as relative estimates of the abundance of excised junctions within each bacterial population. Because multiple primer/probe sets were required to accommodate sequence variation across SCC*mec* backgrounds, excision frequencies were interpreted as relative within-assay estimates rather than absolute copy-number measurements. Furthermore, assay specificity was supported by SCC*mec* type/subtype-specific primer mapping, absence of amplification in no-template controls, and the expected lack of detectable excision signal in SCC*mec* type I.

Reactions were prepared in 10 µL volumes using PrimeTime Gene Expression Master Mix (Integrated DNA Technologies, Coralville, IA, USA) with final concentrations of 0.75 µL each forward and reverse primer for *orfX* and *gyrB*, 0.5 µL each of FAM-labeled *orfX* and HEX-labeled *gyrB* probes, and 1 µL genomic DNA per reaction. Amplification was performed on a Bio-Rad CFX96 Real-Time PCR detection system (Bio-Rad Laboratories, Hercules, CA, USA) using the following cycling parameters: 95 °C for 10 min, followed by 40 cycles of 95 °C for 15 s and 52 °C for 60 s. No-template controls were included on all plates.

Relative SCC*mec* excision frequencies were calculated using the ΔCt method, in which a 10-fold change in template abundance corresponds to approximately 3.32 amplification cycles, reflecting the exponential kinetics of PCR amplification [27,28,29,30,31]. This approach follows the mathematical principles underlying real-time PCR quantification and has been previously applied to measure excision of mobile genetic elements in MRSA. For example, Wu et al. adapted this framework to quantify excision of ACME and SCC*mec* elements in the USA300 lineage [32,35], while Stojanov et al. applied a similar qPCR-based strategy to estimate SCC*mec* excision in the prototypic MRSA strain N315 [26]. Following this established method, the excision frequency was calculated as [32,35]:

$$\mathrm{Excision}\;\mathrm{frequency}=10^{-\frac{\left(C_{t,\mathrm{orfX}}-C_{t,\mathrm{gyrB}}\right)}{3.32}}$$

where $C_{t,\mathrm{orfX}}$ corresponds to amplification of the reconstituted *attB* junction and $C_{t,\mathrm{gyrB}}$ represents the internal normalization control.

### 4.8. Statistical Analysis

All statistical analyses were performed using GraphPad Prism version 10.5.0 (GraphPad Software, Boston, MA, USA). Excision frequencies were log_10_-transformed prior to analysis to stabilize variance and approximate a normal distribution.

Baseline differences in excision across SCC*mec* types were assessed using one-way ANOVA with SCC*mec* type as the categorical factor; the proportion of variance explained is reported as R^2^. Associations between excision frequency and SCC*mec* cassette size were evaluated using simple linear regression of log_10_ transformed excision frequency against element length (bp).

For environmental stress experiments, paired two-tailed *t*-tests were used to compare excision at 37 °C vs. 42 °C and 37 °C vs. desiccation within each strain. Longitudinal trends during continuous culture and sub-lethal oxacillin exposure were assessed by ordinary least squares regression of log_10_ (excision frequency) versus day, performed independently for each strain and condition.

At each sampled time point in the 30-day passage experiment, excision frequencies under continuous culture and oxacillin exposure were compared using unpaired two-tailed *t*-tests. Statistical significance for all analyses was defined as *p* < 0.05.

## 5. Conclusions

In conclusion, SCC*mec* excision was detected across diverse representative MRSA SCC*mec* backgrounds and varied according to both cassette-associated features and environmental context. These findings support SCC*mec* excision as a multi-faceted regulatory process with potential implications for MRSA genomic plasticity resistance acquisition, and dissemination. Future work incorporating multiple strains per SCC*mec* type, together with analyses of recombinase allotype variation, DR-site polymorphisms and strain-level regulatory differences, will be essential for identifying molecular determinants that govern SCC*mec* excision competency across diverse MRSA backgrounds.

## Data Availability

The original contributions presented in this study are included in the article/Supplementary Material. Further inquiries can be directed to the corresponding author.

## Supplementary Materials

The following supporting information can be downloaded at: https://www.mdpi.com/article/10.3390/antibiotics15060555/s1, Figure S1: Relationship between SCC*mec* cassette size and excision frequency under environmental stress (heat and desiccation); Figure S2: SCC*mec* size and excision trajectory during continuous culture; Table S1: Oxacillin susceptibility profiles of MRSA strains by SCC*mec* type/subtype; Table S2: SCC*mec* reference characteristics and genome accessions.; Table S3: Primer and probe sequences used for SCC*mec* excision quantification; Table S4: Primer/probe sets used for each SCC*mec* type or subtype.

## Abbreviations

The following abbreviations are used in this manuscript:

AMR	Antimicrobial Resistance
MRSA	Methicillin-Resistant *Staphylococcus aureus*
MSSA	Methicillin-Susceptible *Staphylococcus aureus*
SCC*mec*	Staphylococcal Cassette Chromosome *mec*
ACME	Arginine Catabolic Mobile Element
*ccr*	Cassette Chromosome Recombinase
HA-MRSA	Hospital-Associated Methicillin-Resistant *Staphylococcus aureus*
CA-MRSA	Community-Associated Methicillin-Resistant *Staphylococcus aureus*
MGE	Mobile Genetic Element
DR	Direct Repeat
*attB*	Bacterial Attachment Site B
attSCC	SCC*mec* Attachment Site
*orfX*	Open Reading Frame X
qPCR	Quantitative Polymerase Chain Reaction
Ct	Cycle Threshold
ANOVA	Analysis of Variance
OLS	Ordinary Least Squares
MIC	Minimum Inhibitory Concentration
CLSI	Clinical and Laboratory Standards Institute
TSA	Tryptic Soy Agar
BHI	Brain Heart Infusion
