# Supplementary material for: Staphylococcal Cassette Chromosome mec (SCCmec) Natural Excision Frequencies and Its Contributing Factors in Variant SCCmec Type Prototypic Strains
Source: Antibiotics (Basel). 2026 May 30;15(6):555. doi: 10.3390/antibiotics15060555 (PMC13296057; doi:10.3390/antibiotics15060555)
Supplement: Supplementary file 1 [file antibiotics-15-00555-s001.zip › antibiotics-4324828-supplementary.pdf]

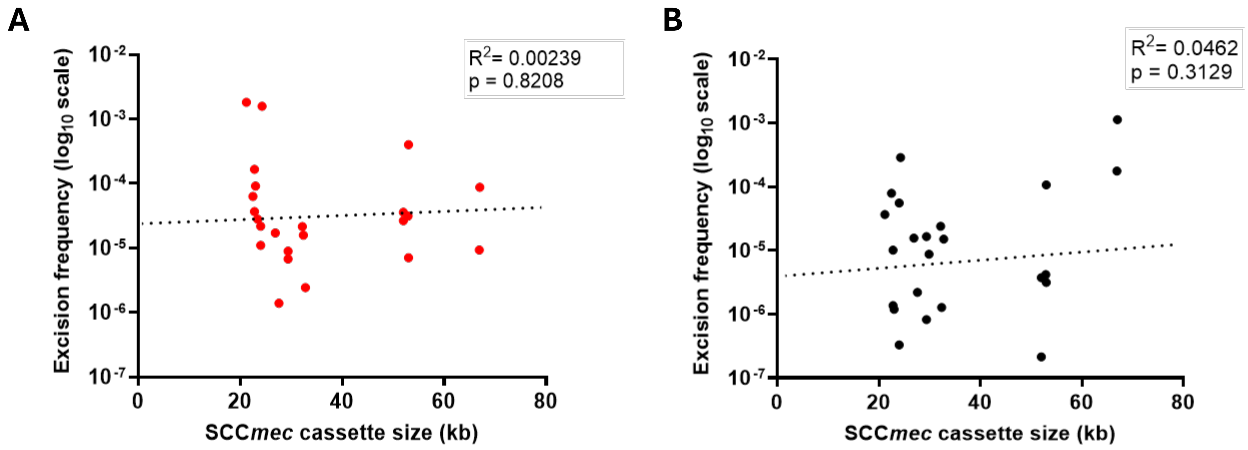

Figure S1: Relationship between SCC*mec* cassette size and excision frequency under environmental stress (heat and desiccation). (A) Linear regression analysis of SCC*mec* cassette size (kb) versus excision frequency (log_{10} scale) under heat stress (42 °C). No significant association was observed (R^2 = 0.00239, p = 0.8208). (B) Linear regression analysis of SCC*mec* cassette size (kb) versus excision frequency (log_{10} scale) under desiccation stress. Cassette size showed no significant relationship with excision (R^2 = 0.0462, p = 0.3129). Each point represents the mean excision frequency from three independent biological replicates for one SCC*mec* type/subtype. Dotted lines indicate the best-fit linear regression.

A

Table. HA-MRSA SCCmec types: size and R^2 (continuous culture)

SCCmec Type	Size (kb)	R^2 (Continuous Culture)
II	52.9	0.896
IIA	53.0	0.831
III	66.9	0.610
IIIA	67.0	0.463

Mean size: 59.95 kb
Mean R^2: 0.700
SD (R^2): 0.200

B

Table. CA-MRSA SCCmec types: size and R^2 (continuous culture)

SCCmec Type	Size (kb)	R^2 (Continuous Culture)
IVa	24.3	0.488
IVb	21.2	0.267
V	27.6	0.483

Mean size: 24.37 kb
Mean R^2: 0.413
SD (R^2): 0.126

C

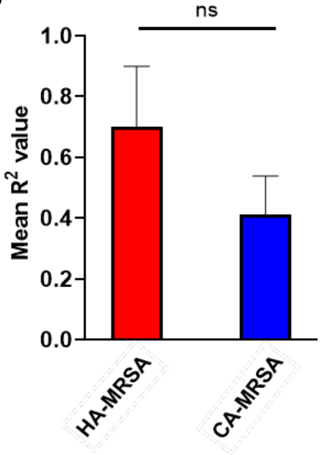

Figure S2: SCC*mec* size and excision trajectory during continuous culture. (A–B) Summary of SCC*mec* cassette sizes and corresponding R^2 values for hospital-associated (HA-MRSA) and community-associated (CA-MRSA) types during 30-day continuous culture. Larger HA-MRSA elements (II, IIA, III, IIIA) generally showed higher R^2 values, reflecting more consistent time-dependent increases in excision, whereas smaller CA-MRSA elements (IVa, IVb, V) showed lower and more variable trajectories. **(C)** Group-level comparison of mean R^2 values for HA-MRSA and CA-MRSA types, showing a higher average R^2 among HA-MRSA elements, although the difference was not statistically significant. Error bars represent standard deviation.

Table S1. Oxacillin susceptibility profiles of MRSA strains by SCC*mec* type/subtype.

SCC*mec* type / subtype	Inhibition zone (mm)	Oxacillin phenotype
I	11.2	I
II	11.0	I
IIA	9.4	R
IIB	8.9	R
IID	10.8	I
IIE	11.4	I
IIb	13.3	S
III	9.2	R
IIIA	10.7	I
IVa	11.1	I
IVb	10.9	I
IVc	13.7	S
IVd	13.2	S
IVE	11.4	I
IVF	11.0	I
IVg	12.4	I
IVh	13.7	S
IVi	11.6	I
IVj	13.1	S
V	11.7	I
VI	13.9	S
VII	13.4	S
VIII	9.4	R
IX	9.8	R

SCC*mec* type / subtype	Inhibition zone (mm)	Oxacillin phenotype
X	12.4	I
XI	13.3	S
XII	10.9	I
XIII	11.8	I

Note: Oxacillin phenotype was determined by disc diffusion. Zone diameters were interpreted according to CLSI criteria, <10 mm, resistant; 11–12 mm, intermediate; ≥13 mm, susceptible. “S” = susceptible, “I” = intermediate, “R” = resistant, according to the CLSI guidelines. Strains classified as resistant or intermediate were eligible for inclusion in prolonged oxacillin exposure assays.

Table S2. SCC*mec* reference characteristics and genome accessions.

SCC*mec* Type	MRSA Strain	SCC*mec* Size (kb)	Accession Number
I	NCTC 10442	34.4	AB033763
II	N315	52.9	D86934
IIA	AR14/0298	~53.0	—
IIB	AR05/0.1345	~28–30	—
IIE	AR13/3330.2	26.9	AJ810120
IIb	05MS-150	~29.0	—
III	85/2082	66.9	AB037671
IIIA	JCSC290	~66–67	—
IVa	CA05	24.2	AB063172
IVb	8/6-3P	21.2	AB063173
IVc	MR108	30.0	AB096217
IVd	JCSC4469	~20–24	AB097677 (partial)
IVE	AR43/3330.1	23.0	AJ810121
IVF	AR43/3636.1	~24–27	—
IVj	JCSC6668	23.0	AB425824
V	JCSC3624 (WIS)	27.6	AB121219
VI	HDE288	22.8	AF411935
VII	JCSC6082	32.4	AB373032
VIII	C10682	32.1	FJ390057
XI	LGA251	29.4	FR821779
XIII	55-99-44	32.7	MG674089

Note: SCC*mec* sizes marked with "~" represent approximate values estimated from published SCC*mec* structural descriptions and available genomic annotations when complete SCC*mec* sequences or accession numbers were not available for the corresponding prototypic strain. Reported sizes are based on previously described SCC*mec* elements of the same type or subtype and are provided to facilitate comparative context across SCC*mec* lineages.

Table S3. Primer and probe sequences used for SCC*mec* excision quantification.

Target	Name	Sequence (5′→3′)	Label
orfX excision site	X1	GAATGAACGTGGATTTAATGTCC	—
orfX excision site	X3-1	GTAACTACGCACTATCATTCAGC	—
orfX excision site	X3-2	GTAACTATGCACTATCATTTAGC	—
orfX excision site	X1-IIE	CTTTGATAAGCCATTCATTCACC	—
orfX excision site	X1-III	ACCTGCTTAAATAATGATAATCAC	—
orfX excision site	X1-IVa	GATAAACAAATTGTTTAAGCTTCC	—
orfX excision site	X1-IVi	GATATTATTTACTTGAAAGACTGC	—
orfX excision site	X1-X	CTAATAATCAAAATTATGTCTGAG	—
orfX excision site	X1-XI	TCAAAAATTTTTATGTGATTGTCC	—
orfX excision site	X1-XIII	CTTCAATAAATTTATAATATGCCC	—
orfX probe	orfX-FAM	/56-FAM/CAACCCGCA/ZEN/TCATTTGATGTGG/3IABkFQ/	FAM
orfX probe	orfX-FAM2	/56-FAM/CAACCCGCA/ZEN/TCATTTGGTGTGG/3IABkFQ/	FAM
gyrB	*gyrB*-F	ATCGACTTCAGAGAGAGGTTTG	—
gyrB	*gyrB*-R	CCGTTATCCGTTACTTTAATCCA	—
gyrB probe	*gyrB*-HEX	/5HEX/CGATGAAGC/ZEN/ATTAGCTGGTTATGC/3IABkFQ/	HEX

Note: Primer/probe sequences are written 5′→3′. Fluorophore/quencher labels are shown where applicable.

Table S4. Primer/probe sets used for each SCC*mec* type or subtype.

Primer pair	SCC*mec* types / subtypes
X1 / X3-1	I, II, IIb, IVb, IVd, IVF, IVj, VI, VII, VIII, IIA, IIB, XII
X1-IIE / X3-1	IIE, IVE
X1-III / X3-1	IVg, III, IIIA, IVc
X1-IVa / X3-2	IVa, V
X1-XI / X3-1	XI, IID, IVh
X1-XIII / X3-1	XIII

Note: Listed primer pairs were used to quantify SCC*mec* excision in the indicated SCC*mec* backgrounds.
